# X-SPEC: a 70 eV to 15 keV undulator beamline for X-ray and electron spectroscopies

**DOI:** 10.1107/S1600577520016318

**Published:** 2021-02-10

**Authors:** Lothar Weinhardt, Ralph Steininger, Dagmar Kreikemeyer-Lorenzo, Stefan Mangold, Dirk Hauschild, David Batchelor, Thomas Spangenberg, Clemens Heske

**Affiliations:** a Institute for Photon Science and Synchrotron Radiation (IPS), Karlsruhe Institute of Technology (KIT), Hermann-v.-Helmholtz-Platz 1, 76344 Eggenstein-Leopoldshafen, Germany; b Institute for Chemical Technology and Polymer Chemistry (ITCP), Karlsruhe Institute of Technology (KIT), Engesserstrasse 18/20, 76128 Karlsruhe, Germany; c Department of Chemistry and Biochemistry, University of Nevada, Las Vegas (UNLV), 4505 Maryland Parkway, Las Vegas, NV 89154-4003, USA

**Keywords:** undulator beamline, soft X-ray, tender X-ray, hard X-ray, *in situ*, *operando*, HAXPES, RIXS, XAS, XES

## Abstract

X-SPEC is a high-flux spectroscopy beamline at the KIT (Karlsruhe Institute of Technology) Synchrotron for electron and X-ray spectroscopy featuring a wide photon energy range. The beamline is equipped with a permanent magnet undulator with two magnetic structures of different period lengths, a focusing variable-line-space plane-grating monochromator, a double-crystal monochromator and three Kirkpatrick–Baez mirror pairs. By selectively moving these elements in or out of the beam, X-SPEC is capable of covering an energy range from 70 eV up to 15 keV. The flux of the beamline is maximized by optimizing the magnetic design of the undulator, minimizing the number of optical elements and optimizing their parameters. The beam can be focused into two experimental stations while maintaining the same spot position throughout the entire energy range. The first experimental station is optimized for measuring solid samples under ultra-high-vacuum conditions, while the second experimental station allows *in situ* and *operando* studies under ambient conditions. Measurement techniques include X-ray absorption spectroscopy (XAS), extended X-ray absorption fine structure (EXAFS), photoelectron spectroscopy (PES) and hard X-ray PES (HAXPES), as well as X-ray emission spectroscopy (XES) and resonant inelastic X-ray scattering (RIXS).

## Abstract 2

X-SPEC is a high-flux undulator beamline for electron and X-ray spectroscopy with an energy range from 70 eV to 15 keV. It offers X-ray absorption spectroscopy (XAS), extended X-ray absorption fine structure (EXAFS), photoelectron spectroscopy (PES) and hard X-ray PES (HAXPES), as well as X-ray emission spectroscopy (XES) and resonant inelastic X-ray scattering (RIXS) for *in vacuo, in situ* and *operando* sample environments.

## 1. Introduction   

X-ray and electron spectroscopies are highly valuable techniques for material characterization, both in fundamental studies as well as for applied systems. Many beamlines worldwide offer a variety of experimental parameters, differing in photon flux, energy range, resolving power, measurement spot size and other parameters. They are often optimized for one or a few particular techniques, including X-ray absorption spectroscopy (XAS) at the near edge (NEXAFS, XANES) or with extended energy range (EXAFS), photoelectron spectroscopy (PES), X-ray fluorescence (XRF), X-ray emission spectroscopy (XES) and/or resonant inelastic X-ray scattering (RIXS).

PES, in particular, is broadly used for the characterization of surfaces in applied materials. The characteristic attenuation lengths (‘inelastic mean free path’, IMFP) of the electrons of a few nanometres make this technique very surface sensitive. At the same time, however, these experiments are often obscured by surface contamination that impedes the measurement of real-world applied systems. It is also difficult to access buried layers or interfaces, which are often of particular interest in applied systems. Thus, in the past few years, hard X-ray PES (HAXPES) has experienced a powerful renaissance in materials science (Woicik, 2016 ▸), which allows the IMFP to be significantly increased [*e.g.* from 1.3 nm for 500 eV electrons to 16 nm for 12 keV electrons in In_2_S_3_ (Tanuma *et al.*, 1994 ▸)]. This development was fueled by a new generation of electron analyzers and suitable hard X-ray beamlines with high resolution and high flux at many synchrotrons around the world (Kobayashi *et al.*, 2003 ▸; Rubio-Zuazo & Castro, 2005 ▸; Gorgoi *et al.*, 2009 ▸; Rueff *et al.*, 2015 ▸; Lee & Duncan, 2018 ▸; Schlueter *et al.*, 2019 ▸). The increased IMFP makes HAXPES also particularly suitable for studies involving higher pressures or surfaces with a thin liquid (*e.g.* water or electrolyte) layer on top. Consequently, a number of beamlines and experimental setups dedicated to ambient pressure and pure gas-phase PES and HAXPES have also been developed (Masuda *et al.*, 2013 ▸; Axnanda *et al.*, 2015 ▸; Weatherup *et al.*, 2016 ▸; Takagi *et al.*, 2017 ▸; Schlueter *et al.*, 2018 ▸; Piancastelli *et al.*, 2019 ▸).

For XAS, *in situ* and *operando* experiments are, traditionally, mostly performed in the hard X-ray range, where experiments can be conducted outside of vacuum under atmospheric pressure, which makes the experimental setups comparably simple. In the past few years, soft XAS, XES and RIXS have been increasingly used for *in situ* and *operando* studies as well, which has been made possible by the development of specialized cells, where, typically, the ultra-high-vacuum (UHV) environment of the analytics is separated from the sample under atmospheric conditions by an ultra-thin membrane (Guo *et al.*, 2002 ▸; Heske *et al.*, 2003 ▸; Fuchs *et al.*, 2008 ▸; Jiang *et al.*, 2010 ▸; Nagasaka *et al.*, 2010 ▸; Blum *et al.*, 2009 ▸; Escudero *et al.*, 2013 ▸; Weinhardt *et al.*, 2013 ▸; Niwa *et al.*, 2013 ▸; Schwanke *et al.*, 2014 ▸; Benkert *et al.*, 2014 ▸; Léon *et al.*, 2019 ▸). These experiments profit from the high sensitivity to the chemical and electronic structure of the soft X-ray spectroscopies and the accessibility of absorption edges of light elements, which are of particular importance for many applied questions (*e.g.* in the fields of batteries, catalysis, and for organic materials).

The central goal of the X-SPEC beamline operated at the KIT Synchrotron is to combine the strengths of HAXPES and soft XAS/XES/RIXS in one instrument, where these techniques can be used on the same spot of one sample, *i.e.* under exactly the same preparation conditions. It is challenging to cover soft and hard X-rays in one beamline, and thus only very few such beamlines can be found worldwide, in particular the ID09 beamline (Lee & Duncan, 2018 ▸) in operation at the Diamond Light Source and EMIL currently coming into operation at the Helmholtz Center Berlin. Both beamlines have a rather similar optical concept, which includes two separate undulator sources that are placed behind each other and are slightly canted. The light from these two undulators is then fed into two separate X-ray branches and focused into a number of endstations. Some of these endstations are used exclusively with soft or hard X-rays, but both ID09 and EMIL have endstations for the use of soft and hard X-rays on the same sample. One of the central design goals of X-SPEC was to keep the beamline layout as simple as possible, to allow quick and easy switching between soft and hard X-rays, to make use of the full length of the straight section in the KARA (Karlsruhe Research Accelerator) storage ring operated at 2.5 GeV, and to cover photon energies as high as 15 keV without any gap between the ‘soft’ and ‘hard’ energy ranges.

Besides HAXPES and soft XAS/XES/RIXS, X-SPEC also offers ‘standard’ techniques, including EXAFS and soft X-ray PES, and two sample stations for experiments under UHV and *in situ/operando* conditions. In the following, we will discuss the design considerations for X-SPEC and the resulting beamline layout, and then give some examples demonstrating the performance of the beamline and its endstations.

## 2. Design considerations and beamline layout   

### 2.1. Undulator source   

The two most central experimental techniques for the X-SPEC beamline are XES (including RIXS) and HAXPES. In the soft X-ray range, the majority of the core-excited states decay via Auger processes, and only a small fraction [*e.g.* 0.03% for S *L*_2,3_ (Krause, 1979 ▸)] of the core holes are filled via fluorescence. This makes XES and RIXS very ‘photon-hungry’ techniques. Likewise, HAXPES experiments also need a high photon flux since the photoionization cross section strongly decreases as a function of photon energy [*e.g.* by more than five orders of magnitude for S 2*p*, when going from 300 to 8000 eV photon energy (Yeh & Lindau, 1985 ▸)]. For the hard X-ray requirements of the beamline, both undulator and wiggler sources would be an option, while the soft X-ray design goals clearly require an undulator. Covering the desired wide energy range with an undulator source is a challenge, and cannot work reasonably with only one fixed period length. One possible solution would be the use of two undulators with different period length, placed behind each other, either inline or slightly canted. This, however, reduces the maximal magnetic length of each device by more than a factor of 2, reducing the achievable flux by a factor of ∼4. It was thus decided to integrate two magnetic structures with different period length into one switchable device, which was developed together with Danfysik. The two magnetic structures have period lengths of 50 mm for soft X-rays and 28 mm for hard X-rays, and a total magnetic length of 3240 mm, making optimal use of one of the long straight sections of the KARA storage ring. A horizontal movement perpendicular to the electron beam allows switching between the two magnetic structures. To achieve the necessary magnetic field, in particular for the structure with short period length, an in-vacuum design is needed, which makes the undulator of the X-SPEC beamline a very unique device (*i.e.* an in-vacuum undulator with two magnetic structures). With a minimal gap of 7 mm, the 50 mm-period undulator offers a minimum photon energy of ∼70 eV in the first harmonic, while the first harmonic of the 28 mm structure can go as low as ∼570 eV. This magnet structure reaches energies above 15 keV using higher harmonics. Thus, the achievable energy range includes the *K* edges from beryllium (*Z* = 4) to krypton (*Z* = 36), the *L*_2,3_ edges from aluminium (*Z* = 13) to thallium (*Z* = 81), and the *M* edges of the heavier elements. A more detailed description of the undulator will be published elsewhere.

### 2.2. Hard X-ray monochromator   

For the hard X-ray range, X-SPEC is equipped with a servo-motor-driven and liquid-nitro­gen-cooled double-crystal monochromator (DCM, FMB Oxford) with Si(111) and Si(311) crystal pairs. To offer a continuous energy spectrum, an important design criterion was an overlap with the maximum energy delivered by the plane-grating monochromator (PGM), with the X-SPEC DCM reaching a minimum energy of 2.03 keV with Si(111). Using the Si(111) and Si(311) reflections, an energy resolution of better than 0.6 eV – very suitable for general-purpose HAXPES experiments – can be achieved up to an energy of 8 keV (see Fig. 3 described in Section 4.2 below). Above 8 keV, high-flux experiments with larger experimental width using Si(311) or Si(111) are still valuable for many applications. To achieve high resolution in this energy range, higher indexed reflections [*i.e.* Si(333), Si(444) or even Si(555)] can be used at (much) lower flux (a few examples will be shown below). A possible later upgrade with a channel-cut post-monochromator space is foreseen in the beamline layout.

### 2.3. Soft X-ray monochromator   

The variety of possible monochromator designs is much higher in the soft X-ray range, with the most prominent solutions being based on the spherical grating monochromator (SGM) (Chen & Sette, 1989 ▸) and the PGM (Petersen *et al.*, 1995 ▸) setups. In terms of the number of optical elements, the SGM setup is superior with only one element (*i.e.* the spherical grating), while the PGM design requires two or three elements (*i.e.* a plane mirror, a plane grating and, in the standard design, a mirror for focusing onto the exit slit). However, the SGM setup requires a variable position of the exit slit and/or entrance slit and can only cover a small energy range with one individual grating. In the PGM design, the exit slit can stay fixed and a broad (the full) energy range can be covered by only one grating. The latter is very important for the wide energy range of X-SPEC. In addition, when using a focusing variable-line-space PGM (FVLS-PGM) (Harada *et al.*, 1984 ▸; Reininger, 2011 ▸), the number of reflections can be limited to two. At the same time, we can achieve a design in which the beams are collinear after passing the DCM or the FVLS-PGM. This is important to fulfill the design goal of simplicity, in which the different beams are close together and within one beam pipe nearly throughout the entire beamline.

The X-SPEC FVLS-PGM (FMB Berlin) has three blazed gratings (DIOS) with different line densities to choose between high resolution and high transmission. To focus on the exit slit, the line density variations across the gratings were optimized in terms of the defocusing and coma terms (Reininger, 2011 ▸). With a fixed 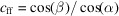 value (with α and β being the grazing incident and exit angle on the grating, respectively), the defocusing term can be kept zero for all energies. The coma term is zero at one energy, but small enough to be negligible for the overall energy resolution of the monochromator at other energies. Table 1 ▸ summarizes the design parameters of the FVLS-PGM.

### 2.4. Focusing optics   

The monochromatic beams from the DCM and FVLS-PGM need to be focused onto the sample in two separate end­stations that will be described below. This is done using three different Kirkpatrick–Baez (KB) mirror pairs. To achieve small spots in both endstations, suitable as a source spot for the entrance-slit-free soft X-ray spectrometers, one KB mirror pair for soft X-rays is positioned close to each of the end­stations. The incidence angle on these mirror pairs is 1.5°, and two different coatings (nickel and rhodium) are used that can be selected by lateral translation of the mirrors. The nickel coating offers high reflectivities below approximately 700 eV, while the rhodium coating is the best choice above this energy. The flat mirrors are bent to elliptical shapes using two bender motors for each mirror, allowing a spot size of <5 µm (vertical) × 90 µm (horizontal). In the hard X-ray range, a small spot size is also needed, and is particularly helpful for HAXPES experiments to operate the electron spectrometer with high-transmission lens modes. A small spot also enables grazing incidence to maximize the photon absorption in the probing volume. With an incident angle of 0.25° and Si/Rh coatings, the ‘hard’ KB pairs are placed 6 times further away from the endstations than the respective ‘soft’ KB pairs. This larger distance is the limiting factor for the smallest possible spot size, and thus the shape requirements for these mirrors are not as strict as for the soft X-ray mirrors. As a result, it is possible to work with the same KB pair for both endstations, and one bender motor for each mirror is sufficient, resulting in a cylindrical shape of the bent mirrors. This enables a hard X-ray beam spot size of <50 µm (vertical) × 700 µm (horizontal) for the first endstation, while the values are approximately 30% larger for the second endstation. Table 2 ▸ summarizes the parameters of the three KB mirror pairs.

### 2.5. Beamline layout   

**Figure 1:**
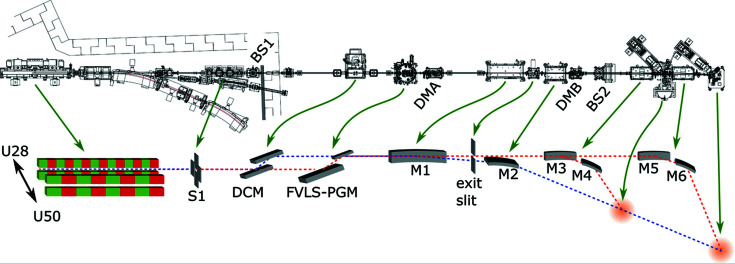
Schematic design of the X-SPEC beamline. The top shows a bird’s-eye view of the actual design drawing, while the bottom shows a schematic of the most important components of the beamline. From left to right: undulator source with two magnetic structures (U28 and U50), slit system (S1), hard X-ray monochromator (DCM), soft X-ray monochromator (FVLS-PGM), hard X-ray mirror M1, soft X-ray monochromator exit slit, hard X-ray mirror M2, and soft X-ray mirrors M3, M4, M5 and M6. The positions of the two endstations in the schematic are depicted with cloudy orange spheres. In the bird’s-eye view, the two beam shutters (BS1 and BS2) and the two diagnostic modules (DMA and DMB) are labeled as well. The soft X-ray beam path is shown in red, the hard X-ray beam path in blue.

The choices discussed above lead to the beamline design sketched in Fig. 1 ▸. X-rays are generated with the in-vacuum double undulator depicted on the left. The U28 and U50 structures are used for energies above and below 580 eV, respectively. The beam from the undulator can be shaped by horizontal and vertical slits (S1) placed in the front-end section to remove undesired light from the up- and downstream bending magnets and reduce the heat load on the optical elements.

For hard X-ray operation, all soft X-ray components are moved out of the beam path. The beam from the undulator (‘pink’ beam) goes directly to the DCM without any pre-mirror, and the monochromatic beam is then focused by mirrors M1 and M2 (using the benders) onto the sample in the first or the second endstation. For soft X-ray operation, all hard X-ray components are moved out of the beam path, and the beam from the undulator is dispersed in energy by the FVLS-PGM and the desired energy is selected by the exit slit. The monochromatic beam is then focused into the first endstation using M3 and M4. With M3 and M4 moved out of the beam and passing through the first endstation, the beam can be focused into the second endstation using mirrors M5 and M6.

Switching between soft and hard X-ray operation is very quick (a few minutes) and requires little to no re-alignment, since only small movements of the optical elements are necessary. Furthermore, the design with the hard and soft X-ray paths in one tube allows mixed operation modes, combining the soft X-ray monochromator with the hard X-ray mirrors, the hard X-ray monochromator with the soft X-ray mirrors, or even the hard X-ray monochromator with hard and soft X-ray mirrors simultaneously to allow for energy filtering, improvements in beamline transmission, spot size, and/or extending the energy range under special operation conditions.

For beamline alignment and diagnostics, two diagnostic modules (DMA and DMB, see bird’s-eye view in Fig. 1 ▸) are placed after the monochromators and after M2, respectively. DMA allows for diagnostics of both the pink and monochromatic beams and contains several fluorescence screens, intensity monitors and beam profile monitors, which are optimized for soft or hard X-rays and pink or monochromatic light. DMA also contains a pink beamstop, preventing the pink beam from traveling further down the beamline and hitting uncooled elements. DMB contains one fluorescence screen, intensity monitors (soft and hard), as well as beam profile monitors (soft and hard). Furthermore, a set of metal foils and filters can be placed in the beam for quick and easy energy calibration or beam attenuation in the hard X-ray range. An additional fluorescence screen is placed right after the front-end section, and, to measure the incoming photon flux, a gold mesh is placed in front of each of the two experimental stations.

## 3. Experimental stations   

X-SPEC is equipped with two experimental stations. In the first station, samples can be studied under UHV conditions with the full suite of experimental techniques (XAS, EXAFS, PES, HAXPES, XES and RIXS). Samples are introduced via a load lock that contains a sample garage and can be connected to sample transport containers for sample transfer without air exposure. The 2.5 × 2.5 cm^2^ sample plates are then transferred into the analysis chamber onto a four-axis manipulator, where samples can be cooled with liquid nitro­gen and heated with an electron beam heater up to 800°C. For further sample preparation steps (*e.g.* ion surface treatments, deposition of metals or organics), the sample can be moved with the manipulator to a preparation chamber. The manipulator is fully motorized and allows for continuous scanning of radiation-sensitive samples under the beam.

For PES and HAXPES experiments, a Phoibos 225 electron analyzer (SPECS) with a 1D delayline detector allows measurement of electrons up to a kinetic energy of 15 keV. Electrons are collected at 90° with respect to the incoming X-ray beam, *i.e.* parallel to the photon polarization vector. For soft XES and RIXS experiments, a high-transmission soft X-ray spectrometer with a resolving power *E*/Δ*E* of 2000 to 4000 and an energy range from 50 to 2000 eV is used, which was developed in-house. A more detailed description of the spectrometer design, performance and first XES/RIXS data will be published elsewhere. For XAS and EXAFS, different detection schemes are possible, including total electron yield using a sample current measurement, partial electron yield detection using the electron analyzer, and (partial) fluorescence yield detection using either a window-less silicon drift detector (Ketek, sensitive from ∼100 eV up to 15 keV) or – with higher emission energy resolution – the soft X-ray spectrometer. All spectrometers and detectors are placed such that they can be used simultaneously on the same spot (provided that the sample is suitably placed in an ‘intermediate’ position).

The second experimental station allows the study of samples at or above atmospheric pressures, *in situ* or even *operando*. The experimental setup is a further evolved version of the SALSA (Solid and Liquid Spectroscopic Analysis) experimental station (Blum *et al.*, 2009 ▸) operated at the Advanced Light Source, Lawrence Berkeley National Laboratory. In such studies, gaseous, liquid or solid samples (or samples consisting of interfaces of these states of matter) are placed behind a thin membrane (*e.g.* ∼100 nm for soft X-ray operation) that separates the sample environment from the UHV of the analysis chamber. The membrane is sufficiently transparent to transmit X-rays of the energy required for the specific XAS, EXAFS, XES and/or RIXS experiments. For soft XES and RIXS, a second soft X-ray spectrometer is used (identical to the one in the first experimental station). XAS and EXAFS experiments can use total electron yield using currents directly collected from the sample or indirectly, *e.g.* from a gold layer deposited on the sample side of the membrane, and/or (partial) fluorescence yield using the soft X-ray spectrometer or a silicon drift detector with a low-energy window (Ketek, sensitive from ∼185 eV up to 15 keV). Furthermore, transmission experiments are possible as well. In the soft X-ray range, this requires a specialized cell, as already used in some experimental setups (Nagasaka *et al.*, 2010 ▸; Schreck *et al.*, 2011 ▸; Schwanke *et al.*, 2016 ▸). For sufficiently high X-ray energies, a beryllium window can be used, and experiments can be performed in a standard ‘in air’ setup, with ionization chambers placed directly behind the experimental station.

Lastly, the second experimental station can be easily removed from the beamline, creating an open port for other types of endstations (*e.g.* for near-ambient-pressure HAXPES).

## 4. First results   

### 4.1. Photon flux   

**Figure 2:**
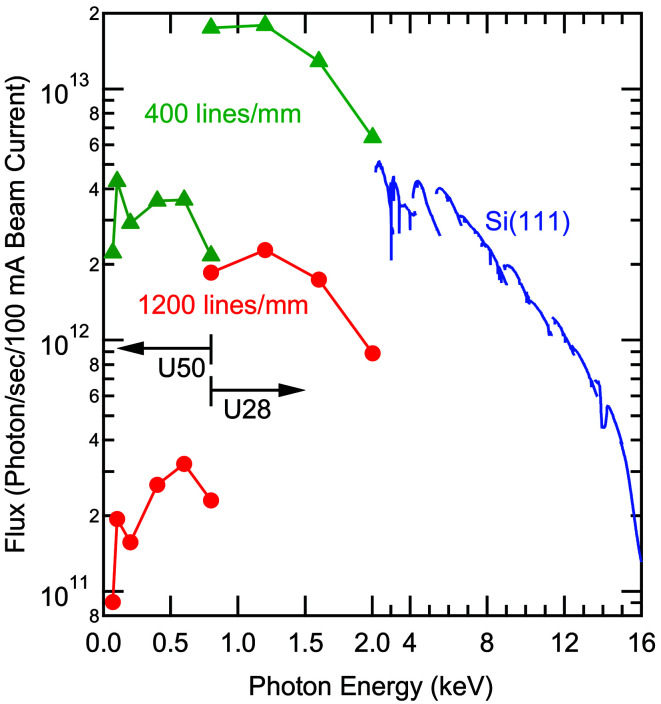
Beamline flux as a function of photon energy. Below 2.0 keV, calculated values for the 400 lines mm^−1^ (green) and 1200 lines mm^−1^ (red) gratings are displayed. In this energy range, the first harmonics of the U50 undulator structure (below 0.8 keV) and of the U28 structure (above 0.8 keV) are used. The curve above 2.0 keV (blue) shows the flux measured with the Si(111) reflection of the DCM and a calibrated photodiode in the second experimental station. For the undulator, the U28 structure with harmonics ranging from third (at ∼2 keV) to 25th (at ∼16 keV) were used.

In Fig. 2 ▸, the flux of the X-SPEC beamline is displayed as a function of photon energy. For soft X-rays, the flux values were simulated using the programs *Wave* (Scheer, 2012 ▸), *Reflec* (Schäfers & Krumrey, 1996 ▸) and *Ray* (Schäfers, 2008 ▸). The exit slit width was set such that the contributions to the energy resolution from focusing of the source and exit slit size were equal. For that, the exit slit size is 21.0 µm for the 400 lines mm^−1^ grating and 10.5 µm for the 1200 lines mm^−1^ grating. High fluxes in the range of 10^12^ photons s^−1^ per 100 mA beam current can be achieved with the U50 undulator and the 400 lines mm^−1^ grating. With the 1200 lines mm^−1^ grating, the flux is still in the 10^11^ photons s^−1^ per 100 mA range. A significant increase of flux (by about one order of magnitude) is achieved when switching from U50 to U28 (at around 600 eV), then reaching more than 10^13^ photons s^−1^ per 100 mA with the 400 lines mm^−1^ grating.

For hard X-rays using the DCM, the beamline flux was measured with a calibrated photodiode mounted in the second experimental station. At slightly above 2 keV, a beamline flux of 5 × 10^12^ photons s^−1^ per 100 mA is measured, which decreases for higher energies to 1 × 10^12^ photons s^−1^ per 100 mA at 12 keV, and then further decreases to ∼1.5 × 10^11^ photons s^−1^ per 100 mA at 16 keV (*i.e.* at an energy that is already higher than the ‘design range’ of the beamline). For energies above ∼12 keV, optimal flux conditions require frequent switching to higher harmonics, and the gain contrast between ‘on harmonic’ and ‘off harmonic’ is not very large if the front-end slits are opened sufficiently. Thus, at these high energies, operating the U28 undulator as a wiggler at the smallest possible gap of 7 mm becomes a practical option. At 15 keV and above, the dominating factor is the reflectivity cutoff of the mirrors. As mentioned above, the design of the beamline also allows additional modes, in which the incident angle on the mirrors can be reduced to increase the flux at these highest energies and further extend the energy range of the beamline.

### 4.2. Energy resolution   

**Figure 3:**
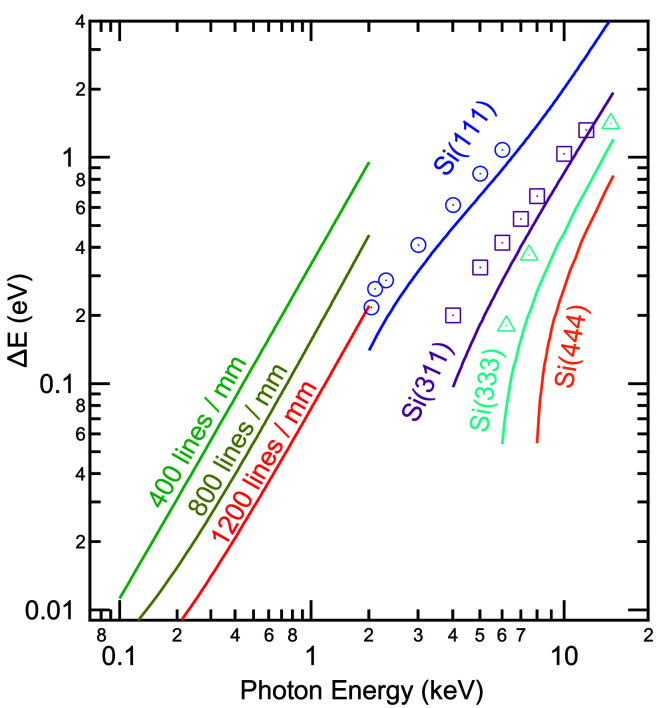
Energy resolution as a function of photon energy. Below 2.0 keV, calculated values for the 400, 800 and 1200 lines mm^−1^ gratings of the FVLS-PGM are displayed. Above 2.0 keV, both calculated (solid lines) as well as experimentally determined (open symbols) values are shown.

Fig. 3 ▸ shows the energy resolution of the beamline as a function of photon energy and monochromator settings. For soft X-rays, the energy resolution for the three different gratings was simulated by using the raytracing program *RAY* (Schäfers, 2008 ▸). Again, the exit slit width was set such that the contributions to the energy resolution from focusing and exit slit size were equal (21.0, 16.0 and 10.5 µm for the 400, 800 and 1200 lines mm^−1^ gratings, respectively). The resolution of the FVLS-PGM follows the known *E* ^3/2^ dependency. For the XES/RIXS experiments, the 400 lines mm^−1^ grating represents a good match in terms of energy resolution (*i.e.* better than 1 eV and as low as ∼15 meV) and high flux needed for these experiments. Depending on the energy range and the requirements of the particular experiment, the 800 and 1200 lines mm^−1^ gratings are a likely choice for XAS and PES experiments. Achievable resolutions with these gratings stay well below 0.1 eV, up to a photon energy of 1 keV, and are only slightly above 0.2 eV at 2 keV.

For the DCM, the energy resolution was calculated using the *Orange Synchrotron Suite* [*OASYS* (Rebuffi & Rio, 2017 ▸)] and the *ShadowOui* software (Rebuffi & del Río, 2016 ▸) and is shown as solid lines in Fig. 3 ▸. In addition, experimental values (open symbols) were determined from gold Fermi edge and 4*f* measurements and include the experimental broadening of the electron analyzer (∼0.15 eV, as estimated from the analyzer settings). Consequently, these values are relevant for HAXPES experiments, while the actual resolution of the beamline (*e.g.* for XAS experiments) will be correspondingly better (*i.e.* Δ*E* will be closer to the theoretical line).

### 4.3. HAXPES spectra   

While HAXPES experiments at kinetic energies of 15 keV (or even above) are very interesting, *e.g.* to maximize bulk sensitivity or probe buried interfaces, most HAXPES beamlines and/or endstations are limited to energies below 12 keV for several practical reasons. First, the photoionization cross sections rapidly decrease for higher photon energies, making it difficult to achieve a good signal-to-noise ratio. At the same time, the monochromator resolution decreases with increasing photon energy and the solutions to overcome this problem significantly reduce the flux, which further decreases the count rate. Finally, it becomes increasingly difficult to construct electron analyzers compatible with very high energies due to the required high voltages, placing high demands on dielectric strength and high-stability power supplies.

At X-SPEC, in contrast, the investigation of applied materials, *e.g.* for energy applications, is of particular importance. For such systems, both very surface sensitive as well as bulk-sensitive probes are required. Thus, one of the design goals was to allow HAXPES experiments at maximum kinetic energy, *i.e.* 15 keV.

**Figure 4:**
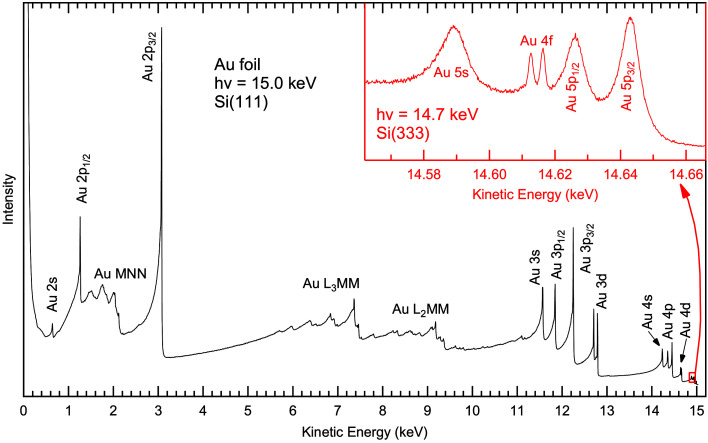
HAXPES survey spectrum of a polycrystalline gold foil, recorded at 15 keV excitation energy using the Si(111) reflection of the DCM. The prominent photoemission and Auger lines are labeled. The red inset on the top right shows the spectral region marked with a small red square in the survey spectrum. It was recorded at an excitation energy of 14.7 keV using the Si(333) reflection of the DCM.

Fig. 4 ▸ depicts the HAXPES survey spectrum of a polycrystalline gold foil taken with an excitation energy of 15 keV. The spectrum covers the full kinetic energy range, from the secondary electron peak at ∼100 eV up to the Fermi edge at 15 keV, and includes all core levels of gold with the exception of Au 1*s*. Despite the low photoionization cross section, the quality of the spectrum is excellent, indicating a successful combination of beamline and analyzer at such high energies (the total measurement time for the spectrum, spanning nearly 15 keV, was 35 min).

While giving high count rates even at 15 keV, the band width when using Si(111) is only suitable for survey (or Auger) spectra at these energies, and higher DCM reflections need to be used to record spectra with high energy resolution. The inset in Fig. 4 ▸ shows the spectral region containing Au 5*s*, 4*f* and 5*p*. While the cross section of Au 4*f* is significantly larger than that of Au 5*s* and 5*p* for typical excitation energies around 1.5 keV, the situation is reversed at 15 keV excitation, since cross sections decrease more rapidly for core levels with higher angular momentum. Furthermore, we point out the low relative intensity of the lines in the small red box, illustrating the small cross sections of the low-lying core levels at 15 keV excitation (and demonstrating the need for adequate photon flux at such high energies).

**Figure 5:**
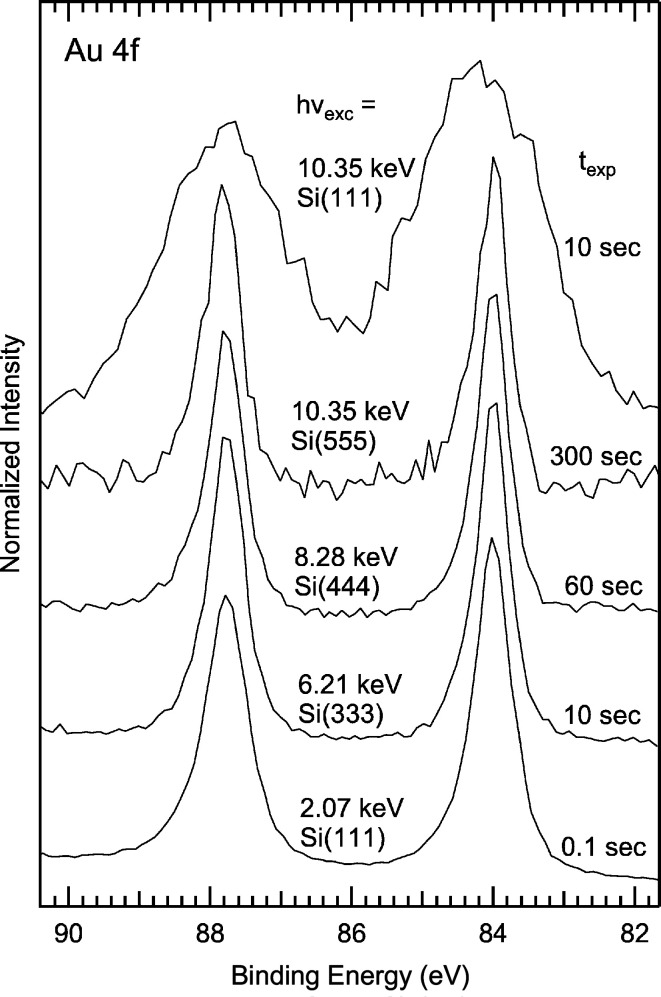
Au 4*f* detail spectra recorded using the ‘Snapshot’ mode of the electron analyzer. Excitation energy, crystal reflection and exposure time are given next to each spectrum.

The Au 4*f* detail spectra shown in Fig. 5 ▸ demonstrate the performance of beamline and electron analyzer at different photon energies. With the high flux of the beamline and the considerably higher cross section for Au 4*f*, a good-quality spectrum can be collected in as little as 0.1 s at an excitation energy of 2.07 keV. Such rapid data collection is possible by using the ‘Snapshot’ mode of the electron analyzer, where the complete energy window is collected in one shot by the ∼100 channels of the 1D delayline detector. With the used settings, the combined energy resolution of the beamline and the analyzer was ∼0.30 eV for the bottom four spectra in Fig. 5 ▸ (and ∼2.3 eV for the top spectrum). We find that for excitation at 6.21 keV using Si(333), spectra with good signal-to-noise ratio can be collected in a few seconds. Good performance is achieved with even higher reflexes, *i.e.* Si(444) and Si(555), and spectra can be collected within a few minutes.

**Figure 6:**
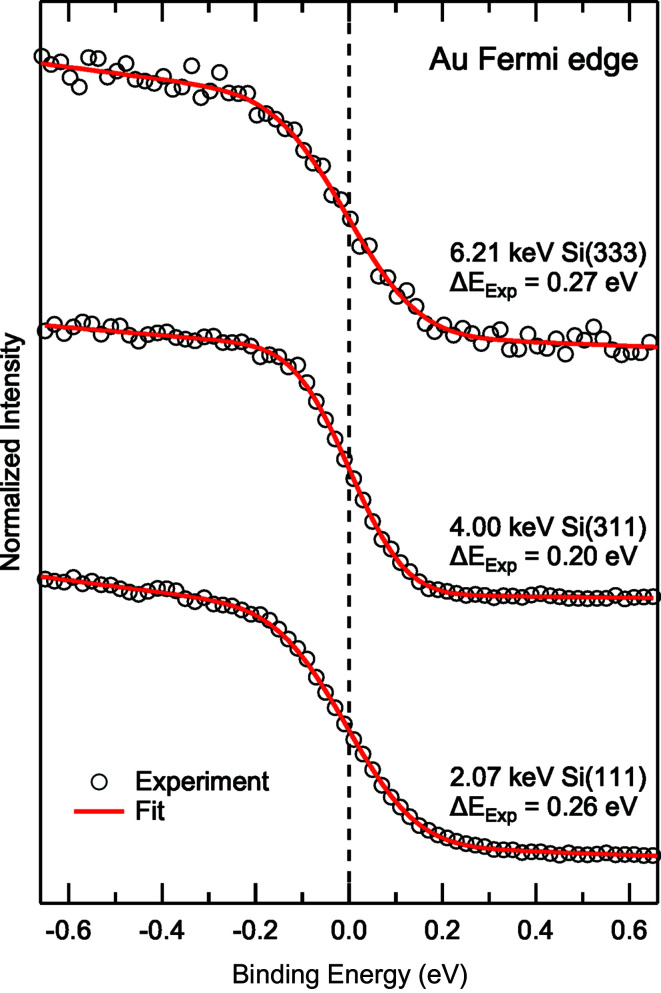
Gold Fermi edge spectra collected at ∼2, ∼4 and ∼6 keV using Si(111), Si(311) and Si(333) reflections for the exciting photon beam, respectively. Open circles show the experimental data and solid red lines the fits. Excitation energies, the used crystal planes, and the combined experimental resolution (beamline plus electron analyzer) as derived from the fit are given for each spectrum.

The spectra of the Fermi edge region of a polycrystalline gold foil collected at excitation energies of 2.07 keV with Si(111), at 4.00 keV with Si(311) and at 6.21 keV with Si(333) are shown in Fig. 6 ▸. These settings give a good energy resolution with sufficient count rates suitable for routine measurements. To derive the experimental resolution, the spectra were fitted using the following analytical function:
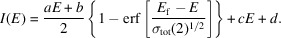


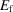
 is the Fermi energy and *a, b, c* and *d* are the fitting parameters for the linear portions to approximate the density of states and the background, respectively. For 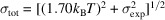, with 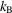 the Boltzmann constant, *T* the temperature and 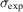 the (Gaussian-type) experimental broadening, an excellent approximation of the convolution between a Fermi function at temperature *T* and a Gaussian broadening is achieved, and the experimental broadening can be derived from the fit.

We find a combined energy resolution (beamline plus electron analyzer) of 0.26 and 0.27 eV for the Si(111) and Si(333) measurements at 2.07 and 6.21 keV, respectively, and a slightly better value of 0.20 eV for the Si(311) measurement at 4.00 keV. Fermi edges with further beamline parameters were collected and the derived values for the experimental resolution are included in Fig. 3 ▸.

## 5. Summary   

The design of the X-SPEC beamline for electron and X-ray spectroscopies using soft, tender and hard X-ray energies is presented. The beamline covers the photon energy range from 70 eV to 15 keV, allows for *in vacuo, in situ* and *operando* experiments. Available spectroscopy techniques include XAS, EXAFS, PES and HAXPES, as well as XES and RIXS. Primary design goals included achieving a high photon flux at good energy resolution and simplicity in terms of beam path to allow easy switching between the different undulator magnet structures, optical elements and monochromators to cover the full energy range. This was achieved by combining a switching in-vacuum undulator with two magnetic structures, a DCM, a FVLS-PGM and three KB mirror pairs for focusing. This design is elegant as it allows integration of all beam paths in one beamline tube and, at the same time, minimizes the number of optical elements and thus maximizes beamline flux.

First example HAXPES measurements were presented that demonstrate the performance of the X-SPEC beamline. In the future development of the beamline, a special focus will be on the construction and integration of specialized environmental cells for *operando* studies of applied materials using X-ray absorption and emission spectroscopies.

## Figures and Tables

**Table:** 

	Substrate	Coatings	Active area	Object distance	Image distance
Pre-mirror	Si	Ni and Au	450 × 40 mm^2^		
Gratings	Si	Au	95 × 15 mm^2^	19000 mm	6500 mm

Line density *n*(*x*) = *a*_0_ + *a*_1_ *x* + *a*_2_ *x* ^2^					

**Table:** 

	*a*_0_	*a*_1_	*a*_2_	*c*_ff_	Blaze angle
Grating 1	400 mm^−1^	0.2548 mm^−2^	5.358 \times 10^{ - 5} mm^−3^	1.5	0.6°
Grating 2	800 mm^−1^	0.3555 mm^−2^	7.754 \times 10^{ - 5} mm^−3^	2.0	0.8°
Grating 3	1200 mm^−1^	0.4308 mm^−2^	9.969 \times 10^{ - 5} mm^−3^	3.0	1.0°

**Table 2:** Parameters of the beamline mirrors M1/M2 is the hard X-ray KB mirror pair, M3/M4 the soft X-ray mirror pair for the first endstation, and M5/M6 the mirror pair for the second endstation. Object distances are measured from the undulator source (M1, M2, M3 and M5) and the exit slit of the PGM (M4 and M6), respectively. For M1 and M2, image distances for both endstations are given.

	M1	M2	M3	M4	M5	M6
Distance to source (mm)	23945	26485	30653	31105	32735	33187
Final shape	Cylindrical	Cylindrical	Elliptical	Elliptical	Elliptical	Elliptical
Object distance (mm)	23945	26485	30653	5605	32735	7687
Image distance (mm)	8055/10555	5515/8015	1347	895	1765	1313
Optically active area (mm^2^)	1300 × 40	400 × 40	400 × 30	200 × 30	400 × 30	200 × 30
Angle of incidence (°)	0.25	0.25	1.5	1.5	1.5	1.5
Coating	Si and Rh	Si and Rh	Ni and Rh	Ni and Rh	Ni and Rh	Ni and Rh
Substrate material	Si	Si	Si	Si	Si	Si
